# Does device matter for inhaled therapies in advanced chronic obstructive pulmonary disease (COPD)? A comparative trial of two devices

**DOI:** 10.1186/s13104-019-4123-5

**Published:** 2019-02-20

**Authors:** Haider Mannan, Soo Wei Foo, Belinda Cochrane

**Affiliations:** 1 Translational Health Research Institute, Western Sydney University, Campbelltown, NSW Australia; 2 School of Medicine, Western Sydney University, Campbelltown, NSW Australia; 3 Department of Respiratory and Sleep Medicine, Campbelltown and Camden Hospitals, Campbelltown, NSW Australia

**Keywords:** Cross over, Inhalation devices, Pulmonary disease, Chronic obstructive, Quality of life, Assessor-blind method

## Abstract

**Objective:**

COPD patients have challenges for effective use of inhalers due to advanced age, fixed airflow obstruction and comorbid medical conditions. Published clinical trials investigate drug efficacy but rarely consider the inhaler device. This trial investigates device efficacy, comparing clinical outcomes for the same medication via two different devices. Our intention was to communicate the results and to critically appraise the study protocol to inform planning of future device comparison research. Subjects with spirometry confirming at least moderate COPD were randomly assigned to inhaler sequence; starting with Accuhaler or metered dose inhaler and spacer (MDI/s). After baseline testing, subjects were assigned to fluticasone propionate/salmeterol xinafoate (SFC) 500/50 mcg twice daily via the first device for 6 weeks’ duration, then changed to the alternate device for the following 6 weeks. Subjects were reassessed in terms of health-related quality of life (HRQL), exercise endurance and lung function after each exposure period.

**Results:**

The recruitment target was not achieved due to unanticipated developments within the pharmaceutical industry, potentially compromising the study’s power. Study outcomes did not differ significantly according to the allocated inhaler device even after adjusting for baseline lung function or inhaler technique. Recommendations for future device comparison protocols are offered.

*Trial registration* Australia and New Zealand Clinical Trials Registry, Current Controlled Trials ACTRN12618000075280, date of registration: 18.01.2018. Retrospectively registered

**Electronic supplementary material:**

The online version of this article (10.1186/s13104-019-4123-5) contains supplementary material, which is available to authorized users.

## Introduction

In chronic obstructive pulmonary disease (COPD), maintenance of therapeutic medication to relieve symptoms is generally delivered via inhalation, in order to preferentially target airways and to lessen systemic impact. Inhaled therapies for COPD were initially assimilated from asthma treatments. Only recently specific medications have been developed for COPD. Many pharmaceutical trials compared inhaled medication efficacy. However, scant published data consider differing inhaler devices in terms of clinical outcomes for COPD.

Inhaler devices may be breath-actuated, such as dry powder inhalers (DPI), or not breath-actuated; a group which includes the nebuliser, Respimat and pressured metered dose inhaler (pMDI). The pMDI’s main limitation is that patients need to coordinate actuation and inhalation. Administering the medication via a volumatic spacer usually overcomes this problem. For breath-actuated devices, coordination is less critical. However, the patient must generate sufficient inspiratory flow to overcome the intrinsic device resistance for effective actuation and drug deposition. Inherent resistance, and hence inspiratory flow requirement, varies by device. Generally, peak inspiratory flow rate (PIFR) greater than 60 L/min is regarded as optimal and less than 30 L/min is insufficient for effective airway deposition via DPI devices [1–7]. For the DPI used in this study, the Accuhaler device (also marketed as Diskus), overall drug deposition and fine particle distribution is relatively stable between these flow rates [1, 3, 8, 9]. Device factors and/or drug formulation are already known to influence clinical efficacy. For example, dosing studies in COPD for tiotropium via the Handihaler and Respimat devices, looking at lung function outcomes, resulted in these products being marketed with daily dosing recommendations of 18 mcg and 5 mcg, respectively [10–12]. Importantly, there is some evidence that a proportion of COPD patients cannot achieve the required inspiratory flow rate to actuate some DPIs commonly used in COPD treatment, with flow rates being lower for older patients and those with more severely impaired lung function [13–15]. Potentially this situation deteriorates further during the state of acute exacerbation (AECOPD), when medication is most needed.

In advanced COPD, respiratory function deteriorates, symptoms are persistent, and many patients develop hyperinflation, gas trapping and respiratory muscle weakness—all factors important in generating inspiratory effort. In patients with severe COPD, a breath-actuated inhaler device might be ineffective, due to limited inspiratory capacity and ergo suboptimal drug delivery. Previous research has demonstrated that drug deposition does occur, even at quite low inspiratory flows, but there may be reduction of the total dose delivered [2, 3, 6]. However, other factors may contribute to device efficacy. In contrast to asthma patients, COPD patients are generally older with increased comorbid medical illness. Commonly associated chronic conditions, such as arthritis, visual, auditory and cognitive impairment may also impact on inhaler capability.

## Main text

### Methods

Subjects were consecutively recruited from Respiratory Outpatients’ Clinic at a tertiary hospital in Sydney. Those eligible were aged more than 40 years and had spirometry in stable state within the previous 12 months that was consistent with moderate to severe COPD. Cognitive function or English language mastery insufficient for informed consent or protocol adherence were exclusion factors, as were contraindication to inhaled corticosteroid (ICS), long-acting beta agonist (LABA) or any of the inhaled medication components of commercially available SFC inhalers. Patients with life-limiting disease of any category, or who were physically too frail, or whose psychosocial circumstances meant that they would be unable to complete the requirements of the study protocol, including attendance for study visits, were excluded. Subjects were ineligible in the setting of AECOPD within the previous 6 weeks and excluded for AECOPD that occurred during the study protocol.

The study was a randomised crossover trial. Subjects completed a recruitment interview after screening for recent AECOPD. At study entry, subjects discontinued current inhaled medications which contained ICS or LABA but were advised to continue all other inhaled medications prescribed by their usual treating doctor. They then performed baseline testing, inhaler technique assessment and education on all trial-related and ongoing inhaler devices. At completion of the baseline visit, subjects were randomised to a sequence of devices and received a prescription for either SFC 500/50 mcg twice daily via Accuhaler or via MDI/s (see Additional file 1: Blinding and randomisation processes). Primary outcome was HRQL assessed by the Saint George Respiratory Questionnaire (SGRQ) score, recalled over the previous month. Secondary outcomes were exercise endurance assessed by 6 min walk test distance (6MWT) in accordance with American Thoracic Society recommendations [16] and lung function measures, such as post-bronchodilator forced expiratory volume in one second (FEV1), residual volume (RV), forced vital capacity (FVC) and maximal inspiratory pressure (MIP). Outcomes were assessed at six and 12 weeks. At each visit, inhaler technique was assessed using checklists available from the Australian Asthma and Respiratory Educators Association. For each device, the subject was assigned a score out of 12 and a rating of optimal, adequate or inadequate, depending on their total score and critical technique errors. At the 6 week visit, subjects received a script for the alternate device and another session of inhaler education. Details of randomization are given in Additional file 1: File S1.

While there were no precedent studies comparing device efficacy for our primary outcome (HRQL), published studies examined during the planning phase of the current study reported statistically significant lung function outcomes for device comparisons with 10–21 participants [17, 18]. Hence, subject recruitment target was set at 40. To impute missing data, multivariate normal imputation was used with 25 imputations [19], allowing repeated measures analysis of covariance (ANCOVA) to estimate the effect of inhaler device exposure on SGRQ score, its component scores and lung function outcomes, after adjusting for baseline FEV1, RV and device technique. Analogous models having interaction between device exposure and technique were fitted. All models were adjusted by period, sequence and subject (sequence). T-tests evaluated the significance for each effect, with ANCOVA results pooled using Rubin’s method [20]. Effect size was estimated by Cohen’s d with small, moderate and strong effects as 0.20–0.49, 0.5–0.79 and 0.8–1.00, respectively [21].

### Results

**Fig. 1:**
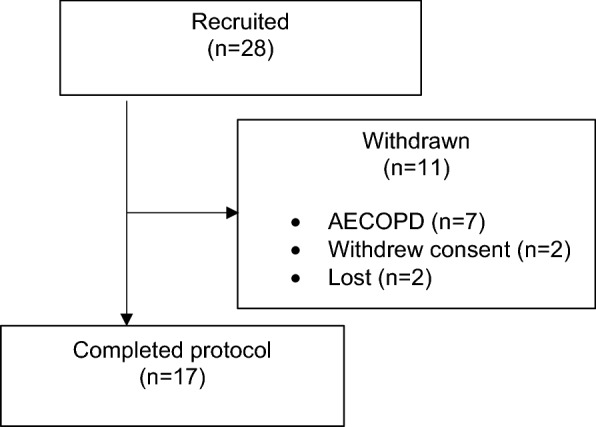
Study recruitment and retention. *AECOPD* acute exacerbation of COPD

Seventeen of 28 subjects completed the study protocol. Eleven subjects discontinued the study; seven due to AECOPD, two withdrew consent and two were lost to follow up. Seven of these subjects were taking MDI/s, three were taking Accuhaler and one failed to fill their prescription. At the time of exclusion due to AECOPD, four subjects were taking MDI/s and three were taking Accuhaler. Figure 1 depicts recruitment and withdrawals.

The subject group had mean age 66 years, slight male predominance (57%) and were mainly former (87%) or current (11%) smokers. They had severe airflow obstruction, moderate gas trapping, substantial symptom burden (as evidenced by SGRQ scores in which a higher score indicates worse quality of life) and significant functional impairment (as indicated by 6MWT results). Baseline characteristics are summarised in Table 1.

**Table 1:** Subject baseline characteristics, n = 27

Mean age (year)	66
Gender	57% male
Mean body mass index, BMI (kg/m^2^)	28.7
Mean smoking (pack year)	49.6
Mean FEV1 (% predicted)	41
Mean FVC (% predicted)	76
Mean FER	0.42
Mean MIP (% predicted)	65
Mean total lung capacity (% predicted)	115
Mean residual volume (% predicted)	161
Mean peak inspiratory capacity (% predicted)	83
Mean gas transfer factor (% predicted)	59
Mean peak inspiratory Flow (L/min)	181
Mean borg dyspnoea score at rest	1.4
Mean 6MWT (m)	297
Mean SGRQ score	49.6

*FEV1* forced expiratory volume in 1 s, * FVC* forced vital capacity, * FER* FEV1/FVC, * MIP* maximal inspiratory pressure, * 6MWT* 6 min walk test distance, * SGRQ* Saint George Respiratory Questionnaire score

Table 2 presents for all outcomes mean difference and effect size by device exposure (Accuhaler versus MDI/s). For the primary outcome (SGRQ score) there was no statistical or clinical difference between the two devices except for impact score which meets criteria for clinical significance. Despite this, a substantial proportion of individual subjects demonstrated a difference in primary outcome and 6MWT for the two devices that was clinically significant (shown in Additional file 2: Figures S2 and Additional file 3: S3).

**Table 2:** Mean difference (effect size) for device exposure (Accuhaler use versus MDI/s) on lung function, quality of life measures and 6MWT, including relevant interactions

Covariate	Dependent variable								
	FEV1 (%)	FVC (%)	RV (%)	MIP(%)	Impact score	Symptom score	Activity score	SGRQ total score	6MWT (m)
Accuhaler (versus MDI/s)	1.47 (0.1438)	2.82 (− 0.2099)	− 2.60 (− 0.0825)	8.10 (0.3656)	− 4.56 (− 0.3667)	− 0.90 (− 0.0653)	− 0.75 (− 0.0559)	− 2.46 (− 0.2189)	8.3 (0.0907)
P value	0.53	0.45	0.77	0.31	0.24	0.86	0.89	0.52	0.74
Inhaler technique (adequate versus inadequate)	2.95 (0.2945)	6.00 (0.5203)	− 2.57 (− 0.0822)	9.87 (0.4625)	− 5.87 (− 0.4643)	− 0.90 (− 0.0654)	− 1.60 (− 0.1195)	− 4.11 (− 0.3615)	3.02 (0.0333)
P value	0.42	0.24	0.85	0.36	0.42	0.86	0.84	0.52	0.95
Accuhaler adequate inhaler technique	2.63 (0.2556)	3.87 (0.3183)	− 15.02 (− 0.4907)	1.41 (0.0690)	− 6.38 (− 0.4897)	− 0.70 (− 0.0523)	− 4.72 (0.3125)	− 5.00 (− 0.4051)	− 9.07 (− 0.1004)
P value	0.67	0.58	0.46	0.93	0.60	0.96	0.69	0.68	0.91

All models were adjusted for period, sequence, subject (sequence) effects to account for design and baseline FEV1, RV and inhaler technique. The t-test was used to test the significance of an individual or interaction effect

*MDI/s* metered dose inhaler and spacer, *FEV1* forced expiratory volume in 1 s, * FVC* forced vital capacity, * RV* residual volume, * MIP* maximal inspiratory pressure, * SGRQ* Saint George Respiratory Questionnaire score, * 6MWT* 6 min walk test distance

### Discussion

This study explored the potential for differing treatment benefits in severe COPD, according to device type, specifically the Accuhaler and the MDI/s. It evolved due to clinical concerns that those COPD patients with more impaired lung function, specifically reduced FEV1, significant hyperinflation or gas trapping, might do worse with breath-actuated devices. In fact, there is evidence that for certain devices suboptimal inspiratory flow rates may result in reduced distribution of medication within the lungs [5, 6]. At the time of the study, in Australia, SFC was the sole commercially available, approved medication product containing comparably dosed inhaled long-acting bronchodilator medication, formulated within two different devices, both breath-actuated and non breath-actuated. New treatment options are now available but commercial factors and regulatory requirements mean that COPD patients may need to master multiple devices. In asthma, having an inhaled treatment regimen comprising multiple devices is associated with increased risk of technique errors in all devices used [22].

Koser et al. compared the Accuhaler and pMDI in terms of safety and efficacy in 247 severe COPD patients, reporting non-inferiority for the pMDI for 2 h post-bronchodilator FEV1 [23]. Drug doses differed marginally between the two devices and the trial used pMDI alone, even though a volumatic spacer is generally recommended. Notably, a recent retrospective database study, evaluating the same devices and medication, using diagnostic codes and prescription records, reported fewer exacerbations and less long-acting antimuscarinic agent (LAMA) use with the pMDI than the DPI at doses 500 mcg/day and 1000 mcg/day, respectively [24].

For SGRQ, there was no statistically significant difference between the two device exposures, even after adjusting for device technique and baseline lung function (FEV1 and RV). This also applied for the secondary outcomes, including SGRQ components (impact, symptom, activity scores), 6MWT and lung function parameters. Provided that the patient is capable of manipulating the device, for the devices tested, treatment efficacy should not differ significantly according to the device. Individual subjects did show clinically different primary outcome with the two devices, which may relate to other factors, such as patient preference, manual dexterity and strength, visual impairment and cognitive dysfunction, which influence inhaler technique and treatment adherence. Hence, treatment choice can rely on these other factors.

### Study strengths

This study’s strengths are the randomised, crossover design, assessor blinding and the use of outcomes reflecting chronic symptom morbidity and functional status. Lung function parameters are the most common measures of treatment efficacy in trials, even though they correlate poorly with symptoms and longitudinal disease progression in COPD [25]. Outcome measures reflecting symptom burden, functional status and quality of life are more meaningful to patients. In addition, we assessed, revised and amended inhaler technique at each study visit. Another strength is to use multiple imputation to handle missing data while performing repeated measures ANCOVA, as it is the recommended approach for such analyses. To better evaluate the validity of our study, we considered both the statistical and clinical significance of our findings [26]. P-values are highly dependent on sample size [27]. Hence, we also calculated effect size, which is independent of sample size [27] and useful for comparing results from different studies [28]. A statistically non-significant result may mean a small sample size was used, while the measured difference in outcome is actually large. However, even using effect size, the two devices did not differ in terms of the main study outcomes. Mixed effects model was not fitted, as the simpler repeated measures ANCOVA will give same results, when all covariates are measured at a single time point.

### Future recommendations

Subject discontinuation was mainly attributable to AECOPD, consistent with other COPD trials. The study protocol specified exposure duration of 6 weeks (12 weeks’ total duration) rather than a briefer period in order that our outcome assessments reflected ICS effect in addition to the LABA effects. A shorter study protocol, whilst it would have been insufficient to fully assess ICS effects, certainly would have reduced participant losses to AECOPD. This latter approach would be preferable for subsequent device comparison studies, particularly if solely comparing bronchodilator products, when each exposure duration could be realistically reduced to 24 h with a consequent improvement in subject retention. The main disadvantage of this approach is losing the effect of practice and inability to assess device technique mastery; both factors being important in clinical practice. Potentially both reduced recruitment and discontinuing subjects contributed to lessen the study’s power to differentiate between the two treatment exposures.

## Limitations

The study also has weaknesses. This research was conducted at a single Australian centre and so the results may not be truly representative of COPD patients. Although the study participants were asked at each study visit about compliance with the study medication, we did not formally record treatment adherence. While the data collected about inhaler device capability is crucial, the data about adherence is less critical in determining the study outcomes, even though this information may have been useful in providing underlying reasons for the results obtained. Importantly, the reliability of assessing compliance differs between the two devices used. Both devices have dose counters, which can count extra doses if dropped or struck, falsely elevating the quantity of doses delivered. In addition for the MDI, actuations can be made into the air (rather than inhaled), which will also artificially exaggerate the number of doses delivered, whether using dose counter or cannister weight to measure this variable. Lastly, for the MDI, protocol adherence required use of a volumatic spacer (as this is known to enhance drug delivery). Unfortunately, there is no effective means of assessing spacer compliance beyond enquiring of the patient.

This is a pragmatic study, reflecting real life clinical practice, in which setting we know that inhaler adherence is generally quite poor. Since both interventions required twice daily dosing regimens, we expected that non-specific non-adherence, such as forgetting to take medication, or not wanting to take medication would not be specific to device and so would impact both intervention periods in the same way. However, an important characteristic of inhaler devices is user-friendliness (or user-appeal). We reasoned that a difference between the devices in terms of this would likely be reflected by a device-specific adherence discrepancy and might translate to a device-specific impact on the study outcomes. However, the foremost limitation was the failure to achieve our pre-specified recruitment target. Several new treatments were released into the Australian market midway through the study. The availability of new, effective inhaled treatments hampered recruitment as potential recruits were reluctant to discontinue these products.

## Additional files

**Additional file 1.** Randomization and blinding.

**Additional file 2: Figure S2.** SGRQ scores post bronchodilator use via Accuhaler and MDI/S.

**Additional file 3: Figure S3.** Subjects’ paired 6MWT results for Accuhaler and MDI/s.

## Abbreviations

COPD: chronic obstructive pulmonary disease
MDI/s: metered dose inhaler/spacer
SFC: fluticasone propionate/salmeterol xinafoate
SWSLHD: South West Sydney Local Health District
HREC: Human Research Ethics Committee
SGRQ: Saint George Respiratory Questionnaire
6MWT: 6 min walk test distance
AECOPD: acute exacerbation of chronic obstructive pulmonary disease
DPI: dry powder inhaler
pMDI: pressurised metered dose inhaler
PIFR: peak inspiratory flow rate
FVC: forced vital capacity
FEV1: forced expiratory volume in one second
MIP: maximal inspiratory pressure
ATS: American Thoracic Society
ICS: inhaled corticosteroid
LABA: long-acting beta agonist
MVNI: multivariate normal imputation
ANOVA: analysis of variance
ANCOVA: analysis of covariance
RV: residual volume
LAMA: long-acting muscarinic antagonist
BMI: body mass index
