# Supplementary material for: Does device matter for inhaled therapies in advanced chronic obstructive pulmonary disease (COPD)? A comparative trial of two devices
Source: BMC Res Notes. 2019 Feb 20;12:94. doi: 10.1186/s13104-019-4123-5 (PMC6383223; doi:10.1186/s13104-019-4123-5)
Supplement: Supplementary file 1 — Additional file 1. Randomization and blinding. [file 13104_2019_4123_MOESM1_ESM.docx]

**Additional File S1** – randomisation and blinding

Randomisation was to a sequence of inhaler devices, commencing with either the Accuhaler or the metered dose inhaler and spacer (MDI/s). A number of sequence allocations totalling the subject recruitment target were concealed within opaque envelopes, 50% indicating a sequence to start with Accuhaler and 50% a sequence to start with MDI/s. At the time of randomisation, an opaque envelope was selected by a study investigator or person outside the trial. Then, a study investigator, who was also a medical practitioner, opened the envelope, noted the randomisation sequence and provided the prescriptions for each of the device exposure periods. The prescription for the initial device was given to the subject, with instructions to fill that prescription, whilst that for the second device was returned to the envelope and kept within that subject’s individual subject file for distribution at the time of commencement of the second device’s exposure period. Subjects were requested not to divulge their device assignment to trial staff at any point during the study protocol.

The research staff responsible for collecting outcome data were not privy to device allocation at the time of randomisation, nor during assessment of the study outcome parameters, which included questionnaire administration, complex lung function testing and assessment of exercise endurance. At study visits, instruction about inhaler technique was provided for each of the comparator inhaler devices and for any additional ongoing inhaler devices. Hence, the investigator staff responsible for collecting outcome data were blinded as to treatment assignment, although subjects, and the investigators involved in provision of prescriptions, were not blinded. No breaches of allocation concealment were reported for the duration of the trial.
