# Supplementary material for: Does device matter for inhaled therapies in advanced chronic obstructive pulmonary disease (COPD)? A comparative trial of two devices
Source: BMC Res Notes. 2019 Feb 20;12:94. doi: 10.1186/s13104-019-4123-5 (PMC6383223; doi:10.1186/s13104-019-4123-5)
Supplement: Supplementary file 2 [file 13104_2019_4123_MOESM2_ESM.docx]

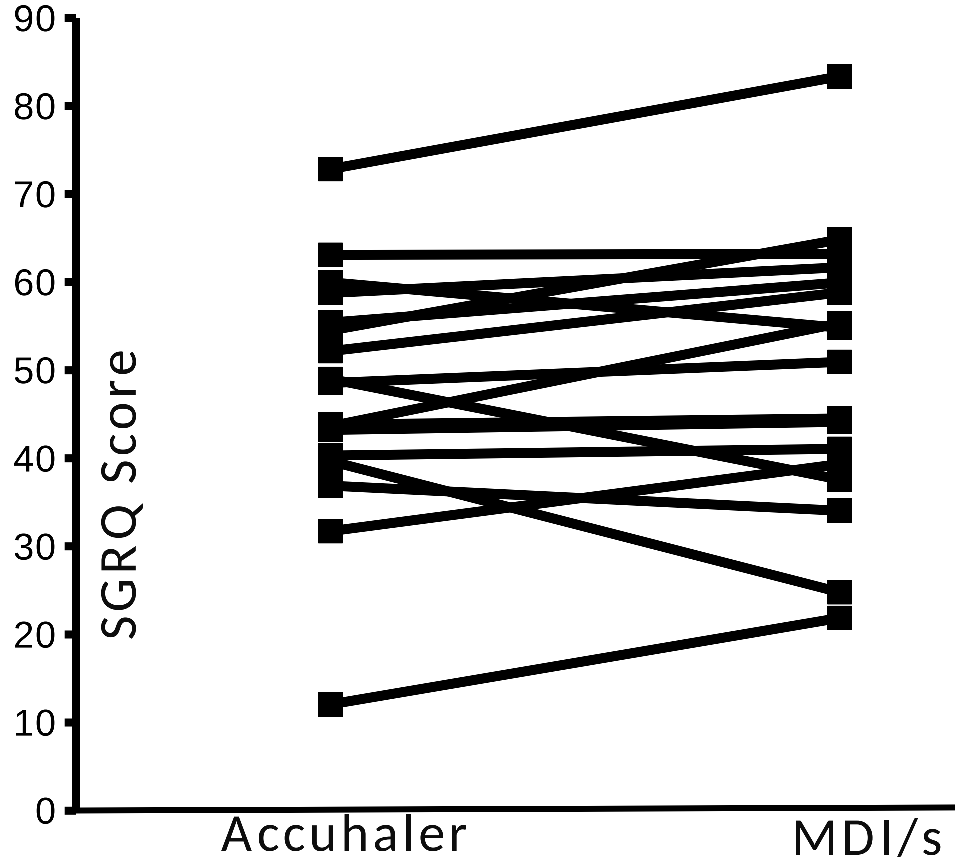

SGRQ Fig 2
