# Supplementary material for: Does device matter for inhaled therapies in advanced chronic obstructive pulmonary disease (COPD)? A comparative trial of two devices
Source: BMC Res Notes. 2019 Feb 20;12:94. doi: 10.1186/s13104-019-4123-5 (PMC6383223; doi:10.1186/s13104-019-4123-5)
Supplement: Supplementary file 3 [file 13104_2019_4123_MOESM3_ESM.docx]

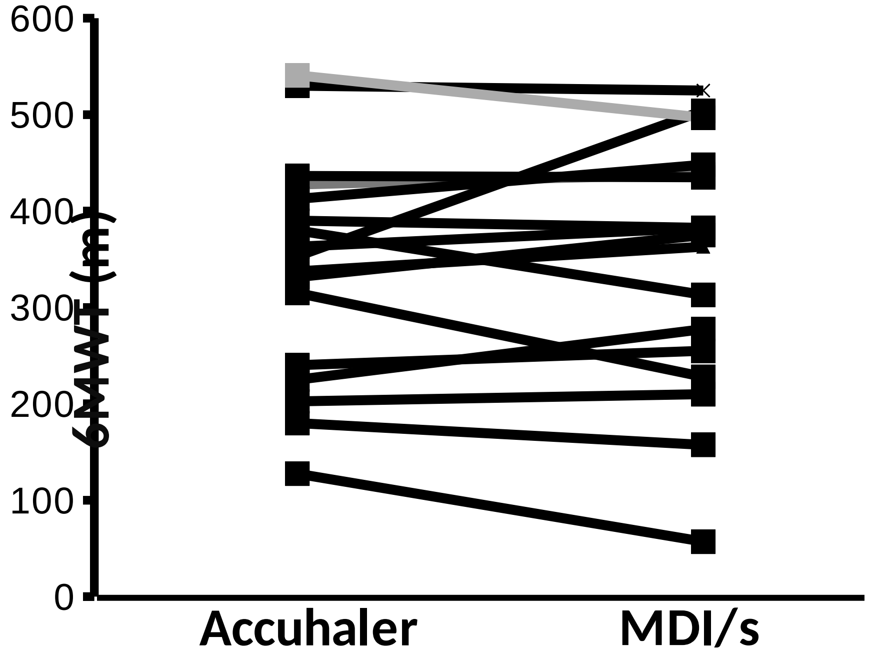

6MWT (m)
600
500
400
300
200
100
0
Accuhaler
MDI/s
